# Supplementary material for: Learning curve and surgical outcome of robotic assisted colorectal surgery with ERAS program
Source: Sci Rep. 2022 Nov 29;12:20566. doi: 10.1038/s41598-022-24665-w (PMC9709162; doi:10.1038/s41598-022-24665-w)
Supplement: Supplementary file 1 — Supplementary Information 1. [file 41598_2022_24665_MOESM1_ESM.zip › LSC Raw data-Ñ[▒K/149/N_ñw╝╨░O▒Kñσ.pdf]

W102 / W102

# 臺 中 榮 民 總 醫 院
## 手術室護理記錄

[redacted]

## 基本資料

- 室別： 開刀房-06室，手術科別： CRS，手術類別： 常規，日期： 2022/01/06
- 進入時間： 1110 ； 麻醉開始時間： 1115 ； Time Out時間： 1230 ； 劃刀時間： 1231 ； 手術結束時間： 1010 送出時間： 1615 ；
- 麻醉方式： GE
  診斷：RO Malignant tumor of A-colon
  手術名稱：Laparoscopic right colectomy and anastomosis
  手術局部用藥：nil
  術前預防性抗生素：
  - 抗生素名稱一： Veterin 1GM IVD/由麻醉護理執行
  - 時間： 1225
  - 上次簽章: 彭馨慧

  ，追加預防性抗生素：
- 手術醫師： 蔣鋒帆，楊曙榮，林志安，蔡濬樟
- 麻醉醫師： VS 賴慧卿
- 刷手護士A ： 彭馨慧，時間： 1110，接替刷手一： 許晏妮，時間： 1130
  流動護士A ： 蔡易軒，時間： 1110，接替流動一： 彭馨慧，時間： 1200

## 皮膚狀況

A-V Shunt： 無
術前： 完整
  記錄者： 上次簽章: 彭馨慧
術後： 完整
  記錄者： 彭馨慧

## 手術準備

手術臥式 單一手術臥式

- 
  - 手術臥式： 仰臥
  - 使用附件： 溫毯 38 ℃，頭圈
  - 皮膚準備： 10%Aq Betadine，Hibiscrub，2% Chlorhexidine
  - 電燒： 使用電燒: 電極片位置： 右大腿，記錄者： 上次簽章: 彭馨慧

止血帶T： 無

## 植入物

- Ethicon ligaclip/20釘/LOT V94K55/pelvic
  Ethicon 75mm/貳釘/colon/LOT U40A3W
  Surgiwrap 100*130mm/pelvic/壹

，記錄者: 上次簽章: 彭馨慧

## 標本送檢

- 細菌培養 無
- 細胞學 無
- 冰凍切片 無
- 病理標本
  組織名稱一：A-colon
- 截肢 無

標本交付 標本： 壹 袋，標本： 零 盒，標本： 零 瓶，細胞學： 零 管，Culture Tube： 零 管，被交付醫師： R 林志安，

- 記錄者： 彭馨慧
- 時間： 1610

## 傷口術後數料

傷口數料：　　　　　　　　　　　　傷口數料： 無

- 請於圖上標示部位：

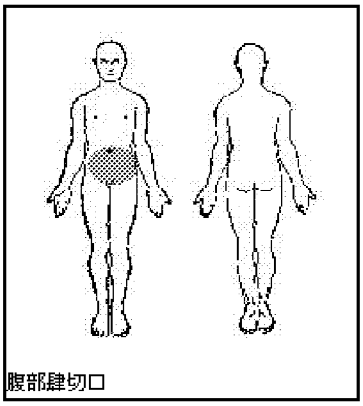


- 傷口數料： menpor覆蓋

## 導管

| 導管 | 天數 | 導管資訊 | 插入日期 | 插入時間 | 更換日期 | 拔除日期 | 拔除時間 | 拔除原因 | 預計拔除/更換 | 預計拔除/更換日期 |
|---|---|---|---|---|---|---|---|---|---|---|
| Foley(1) | 1 | 號碼:16 Fr.; 部位:尿道; | 2022/01/06 | 1200 | | | | | | |
| Sump NG Tube(1) | 1 | 號碼:16 Fr.; 部位:胃; 註記:固定55公分; | 2022/01/06 | 1140 | | | | | | |
| A-line (1) | 1 | 號碼:20號' ; 部位:左手; | 2022/01/06 | 1150 | | | | | | |

## 用物計數

- 手術中出血量： 少量 c.c.
- 手術中尿量： 300 c.c.
-
    - 其它： 無

用物計數： 多切口同時縫合

- 1.器械： 正確，2.縫針： 正確，3.刀片： 正確，4.紗布： 正確，5.腹部墊： 正確，6.紗球： 未使用，7.PEANUTS ： 未使用，8.紗條： 未使用，9.絲帶： 未使用，10.針頭： 未使用，11.Vessel loop： 未使用，12.Boots： 未使用，13.Jelco： 未使用，14.棉條： 未使用，
    - 第一次：
        - 刷手護士 ： 許晏妮
        - 流動護士： 上次簽章: 彭馨慧
        - 時間： 1520

    ,
    - 第二次：
        - 刷手護士 ： 許晏妮
        - 流動護士： 上次簽章: 彭馨慧
        - 時間： 1530

    ,
    - 第三次：
        - 刷手護士 ： 許晏妮
        - 流動護士： 上次簽章: 彭馨慧
        - 時間： 1540

## 備註

備註： NIL

- 記錄者： 上次簽章: 彭馨慧
- 時間： 1530

填寫護理人員 彭馨慧

正式摘要

W102 / W102

doctorRank:B
